# Vocal Cord Palsy Post Chemoradiation in Head and Neck Cancer: Challenges After Cure

**DOI:** 10.1007/s12070-025-05473-w

**Published:** 2025-05-13

**Authors:** Abhilash Goyal, Kirti Khandelwal

**Affiliations:** 1 Department of General Surgery, AIIMS Guwahati, Guwahati, Assam India; 2 Department of Head and Neck Oncology, Tata Memorial Hospital, Mumbai, Maharashtra 400012 India

**Keywords:** Vocal cord palsy, Radiation induced vocal cord palsy, Head and neck cancer, Chemotherapy induced vocal cord immobiliity

## Abstract

Chemoradiotherapy plays an important role in treatment of head and neck cancer. Though it enables cure, it is also associated with range of side effects. Vocal cord palsy is rare, under reported, but can be life threatening. To understand how chemo radiation impacts the vocal cords is critical for effective case management and treatment planning. We conducted a comprehensive electronic search across Cochrane, EMBASE, and PubMed to identify relevant studies. A Google search was also performed using the keywords to ensure the inclusion of all articles reporting vocal cord palsy following treatment for head and neck cancer. Out of 65 articles, 24 were included for analysis to understand the pathophysiology of radiation- or chemotherapy-induced vocal cord paralysis. Additionally, eight articles that reported cases of vocal cord palsy in head and neck cancer other than nasopharyngeal and oropharyngeal were reviewed in detail case wise. The incidence of radiation-induced neuropathy ranges from 1 to 9%. The cranial nerves most commonly affected are the vagus, trigeminal, spinal accessory, oculomotor, abducens, optic, and hypoglossal nerves, with the damage being irreversible. Most cranial nerve neuropathies occur following radiation to the skull baseor neck. In head and neck cancer, nasopharyngeal cancer is most frequently associated with vocal cord palsy after radiation, typically occurring 2 to 10 years posttreatment. Bilateral involvement is more common, though in cases of unilateral involvement, the left vocal cord is more often affected. A detailed review of radiationinduced vocal cord palsy in laryngeal and hypopharyngeal cancers has been conducted. It is essential to rule out recurrence before attributing the palsy to radiation. Chemotherapy-induced vocal cord palsy, which is usually bilateral, typically occurs during chemotherapy and often reverses upon discontinuation of the causative agent. Vocal cord palsy post chemo radiation in head and neck cancer represents a journey of from cure to challenge. It may be a silent struggle for many, but can be very serious for some. Hence, it should not be ignored. Thorough documentation of vocal cord status post treatment is important in follow up for head and neck cancer. Understanding the effects of organ preservation strategies helps in making more informed decisions for patients and plan appropriately.

## Introduction

Radiotherapy and chemotherapy play a crucial role in the treatment of head and neck cancer. However, these treatments are associated with a range of side effects. Although vocal cord paralysis is not commonly reported, it can significantly affect speech, swallowing, and breathing. This review focuses on vocal cord palsy (VCP) resulting from radiation and chemotherapy used to treat head and neck cancers, with particular emphasis on radiation therapy for laryngeal and hypo pharyngeal cancers. There is a growing shift towards organ preservation in the treatment of these cancers, making the assessment of vocal cord mobility an essential part of patient evaluation. A thorough understanding of how chemo radiation impacts the vocal cords is critical for effective case management and treatment planning.

## Methods

### Search Strategy

IWe conducted a comprehensive electronic search across Cochrane, EMBASE, and PubMed to identify relevant studies for the mini review. The search terms used included: radiation-related vocal cord palsy, radiation-induced vocal cord paralysis, radiation-induced vocal fold immobility, radiation-induced vocal cord immobility, radiation-induced vocal cord palsy, radiotherapy-induced vocal cord palsy, and late vocal cord complications of radiotherapy, chemotherapy induced vocal cord palsy, chemotherapy induced vocal cord immobility in the context of head and neck cancer. A Google search was also performed using the same keywords to ensure the inclusion of all articles reporting vocal cord palsy following treatment for head and neck cancer. No data restrictions were applied. After removing duplicates, the authors independently reviewed the articles. Of the 65 articles retrieved, 24 were included for analysis to understand the pathophysiology of radiation- or chemotherapy-induced vocal cord paralysis. Additionally, eight articles that reported cases of vocal cord palsy in tumors other than nasopharyngeal and oropharyngeal were reviewed in detail on a case-by-case basis.

## Discussion

### Causes of Vocal Cord Palsy

The most common etiology for both unilateral and bilateral cord immobility is surgery. Malignancy ranks the second. Additionally, idiopathic causes are responsible for unilateral immobility, while intubation for bilateral immobility [1]. Incidence of radiation induced neuropathy ranges from 1 to 9% [2, 3]. Most commonly affected cranial nerves are the vagus, trigeminal, spinal accessory oculomotor, abducens, optic, and hypo glossal nerve [4], which is irreversible. Majority of the cranial nerve neuropathies occur after radiation to skull base or neck. The pons and medulla are only separated from the nasopharynx by the basi occiput. Regarding head and neck cancers, vocal cord paralysis is more commonly observed following radiation in patients with nasopharyngeal cancer [5]. It is associated with multiple cranial nerve palsies and bilateral laryngeal involvement [6, 7]. Interval between radiotherapy and onset of symptoms indicating brainstem radio necrosis (blindness, multiple cranial nerve palsies, hemiparesis, hemi sensory deficit, and ataxia) usually vary from 3 to 26 months but can be as late as 35 years [8]. Table 1 illustrates the literature relating to vocal cord palsy in non nasopharyngeal non oropharyngeal head and neck cancers.

- Berger et al. [4] described four distinct zones of irradiation reactions in the brainstem of a patient with oropharyngeal carcinoma. The most anterior zone, closest to the radiation field, exhibited necrosis accompanied by fibrosis, exudation, vessel wall degeneration, and complete demyelination. Posterior to this zone, where radiation exposure was lower, there was intense reactive change with dilated blood vessels and partial demyelination. Further posteriorly, a zone of mild reactive changes was noted, followed by a region of normal brain tissue that had received the least radiation exposure. Why does radiation neuropathy occur?

**Table 1:** illustrates review of literature on vocal cord motion impairment after radiotherapy in non nasopharyngeal non oropharyngeal head and neck cancer

Study	Age at presentation/Gender	Subsite	Cord involved	Mean duration	RT planning
Berger and Bataini, [9]	4/25	1- supraglottic, 1- hypopharynx, 1-MUO,1-uvula	Isolated vagus nv involv	12–85 months	Conventional 2D RT
Stern et al. [10]	58 yr/M	Ca glottis cT1N0	Left VC palsy Right VC- Slight mobility	21 yrs	Midplane full tumor dose of 72 Gy in 36 fractions (200 cOy × 6 per week) by parallel opposing portals measuring 6 × 6 em on a cobalt 60machine
Stern et al. [10]	30 yr/F	Thyroid Ca-Pap	B/L VC palsy(Rt VC-injured in surgery)	34 yrs	Tumor dose of 2,000 R by parallel opposing portals measuring 8 × 10 em to each side of the neck and another tumor dose of 2,000 R by an anterior portal measuring 6 × 10 em to the sternal region
Prepageran and Raman [11]	77 yr/M	Laryngeal Ca	B/L VC-median position	15 yrs	
Maruyama et al.[12]	67 yr/F	Laryngeal Ca	B/L VC-median position	28 yrs	Parallel opposing portal technique dose of 6000R using X-ray betatron
Crawley and Sulica [13]	5–40,41,56,68/M and 70/F	Hodgkin lymhoma	3 left Vc Plasy, 2 B/L VC palsy	20–27 yrs	
Singh [14]	73 yr/M	Glottic Ca	B/L VC-median position	4 yrs	Not mentioned

Radiation neuropathy is typically progressive and irreversible [15]. Treatment related risk factors are total radiation dose, dose per fraction, surgery, chemotherapy. It develops months to years after the completion of therapy. The latency period is inversely related to the radiation dose [9]. Patient related risk factors include age, obesity, co morbidities, and preexisting neuropathies [16]. Peripheral nerves are regarded as relatively resistant to radiation; yet, cases of delayed radiation-induced peripheral neuropathy have been reported in the literature since 1966 [17].

#### Pathogenic Mechanisms

Nerve damage results from a combination of direct axonal injury and demyelination leading to *focal nerve damage*, indirect connective tissue damage around nerves leading to *scarring*, and *ischemia* because of interruption of small vessels and local capillaries [15].Schwann cells are affected, suffering a permanent depletion which may contribute to functional impairment [17]. This damage accumulates to produce symptoms of neuropathy over the course of years.

Radiation fibrosis has three stages: a prefibrotic phase involving endothelial cells, a fibrotic phase with fibroblast deregulation and active matrix deposition, and a *late fibrotic-atrophic* phase with retractile fibrosis and apoptosis [18]. Radiation induced neck fibroses in the vicinity of nervesmay lead to the mechanical compression of the nerves, resulting in permanent functional change [19]. Huang and Chu mentioned retro parotid space fibrosis as the cause of palsies of cranial nerves IX–XII [20].

Vocal cord palsies can occur because of neuropathies post radiation or crico arytenoid joint changes. Radiation-induced changes to the crico arytenoid joint may also lead to fixation and/or impaired mobility of the vocal fold. Laryngeal electromyography is the problem solving tool which helps in differentiating neural injury from crico arytenoid joint ankylosis.

### Differentiate Between RLN Palsy and Cricoarytenoid Joint Fibrosis?

- In recurrent laryngeal nerve palsy there will be adducting movements of the vocal cords due to the innervation of the superior laryngeal nerve but the cord might appear thin and wasted.
- In cricoarytenoid joint fixation, the joints will be ankylosed and fixed during palpation.

### Presentation?

Presentation usually varies.

- HHoarseness of voice.
- Vocal fatigue.
- Breathing difficulty.
- Stridor.
- Cough.
- Aspiration.
- Recurrent chest infections.

### Which Cord Affected More?

Bilateral cord palsies are majorly seen in all post radiation setting. Phonation is near normal in these cases, but they present with respiratory distress which can be life threatening. Nasopharyngeal cancers or tumors near skull base typically present with bilateral cord palsies. In cases of unilateral vocal cord palsy, the left cord is more commonly affected. If radiation leads to local hypovascularity, fibrosis, and contracture, creating a "first hit" that increases vulnerability to viral-associated or idiopathic neuropathy, due to the greater length and exposure of the left-sided nerve there is a predisposition to left-sided vocal cord palsy. Unilateral cord palsies present with hoarseness, voice fatigue, cough or aspiration.

### Direct Laryngoscopy Findings?

Direct laryngoscopy reveals a narrowed laryngeal inlet, with the vocal cords appearing fixed in a para median position and a very narrow glottic chink in cases of bilateral nerve involvement. Palpation of the arytenoid shows it to be hard and fixed, with immobility of the crico arytenoid joint. The laryngeal framework, including the aryepiglottic fold, ventricles, and false cords, appears firm and fibrosed, with pale, atrophic laryngeal mucosa.

### Imaging Signs

BECKER’s imaging [21] changes post radiotherapy- atrophy of thyroarytenoid muscle, enlarged ventricle, ipsilateral enlargement of the pyriform sinus, and low attenuation of muscle on a Ccomputed tomography (CT) scan images seen at the level of the true vocal cord.

### Distinguish Between Tumor Recurrence and Radiation Myelopathy

- It is imperative to distinguish between tumor recurrence and radiation myelopathy. Tumor recurrences are most common within first two years of treatment while radiation myelopathy lesions occur from 2 to 10 years. However, tumor recurrence, metastasis or second primary is far more likely to occur [4]. So patients should be very carefully followed.

### Does IMRT Era Will Make a Difference?

Most of the studies involved 2D radiotherapy technique. Effects of intensity modulated radiotherapy needs to be reported and explored in detail.

### Effect of Chemotherapy on Vocal Cord Palsy?

Peripheral neuropathy is a known adverse effect of several classes of chemotherapeutic agents, including vinca alkaloids, taxanes, and platinum agents [22]. Chemotherapy-induced neuropathy is primarily sensory, length-dependent, and peripheral. M otor neuropathy is less common and is often associated with anti mitotic agents, such as vinca alkaloids and taxanes [23].

Chemotherapy-induced vocal fold motion impairment (CIVFMI) was reported in 1971 [24]. Vincristine is the most common chemotherapeutic agent reporting vocal cord palsy. The majority were paediatric population who underwent treatment for Acute Lymphoblastic Leukemia (ALL) [25]. Most cases of CIVFMI resolved with the cessation of therapy. Talmor et al. [26] reviewed literature of chemotherapy induced vocal cord palsy (VCP). Platinum and taxanes are more commonly used in head and neck cancer. Cisplatin [27], carbo platin and paclitaxel [28] and oxali platin [29] have been reported as single case. Symptom duration is variable, ranging from 7 to 420 days with a mean of 105.9 days. Dysphonia is the most common presenting symptom followed by stridor, which was present in about 40% of cases. Dysphagia has a similar prevalence rate to stridor in CIVFMI in the pediatric age group. More than half of the cases had bilateral vocal cord involvement. In cases of unilateral palsy, left was more common than right [26].

## Conclusion

Vocal cord palsy is a silent but serious struggle after radiation in head and neck cancer. Though it is rare, but, still can be life threatening. Having said that, it has a variable and delayed presentation. Bilateral cord involvement is more common. Choosing patients wisely for organ preservation is imperative. History of head and neck radiation in past should be considered while planning treatment in recurrent cases. A stringent follow up with detailed documentation of vocal cord status is required in all head and neck cancers.

## Declarations

### Human or Animal Participants

Research does not involve new human participants. It is just reviewing published reports. Hence, consent for the present review is not required.
